# Coupling Analysis on Microstructure and Residual Stress in Selective Laser Melting (SLM) with Varying Key Process Parameters

**DOI:** 10.3390/ma15051658

**Published:** 2022-02-23

**Authors:** Peiying Bian, Chunchang Wang, Kewei Xu, Fangxia Ye, Yongjian Zhang, Lei Li

**Affiliations:** 1 Xi’an Key Laboratory on Intelligent Additive Manufacturing Technologies, Shaanxi Key Laboratory of Surface Engineering and Remanufacturing, Xi’an University, Xi’an 710065, China; bobal@163.com (K.X.); yfx324@163.com (F.Y.); zhangyongjian@mail.nwpu.edu.cn (Y.Z.); lilei1634@sina.com (L.L.); 2 Shaanxi Tyon Intelligent Remanufacturing Co., Ltd., Xi’an 710018, China; susanzhaoyue@163.com

**Keywords:** selective laser melting (SLM), process parameters, microstructure, residual stress

## Abstract

With the application of Selective Laser Melting (SLM) technology becoming more and more widespread, it is important to note the process parameters that have a very important effect on the forming quality. Key process parameters such as laser power (*P*), scan speed (*s*), and scanning strategy (μ) were investigated by determining the correlation between the microstructure and residual stress in this paper. A total of 10 group 316L specimens were fabricated using SLM for comprehensive analysis. The results show that the key process parameters directly affect the morphology and size of the molten pool in the SLM deposition, and the big molten pool width has a direct effect on the larger grain size and crystal orientation distribution. In addition, the larger grain size and misorientation angle also affect the size of the residual stress. Therefore, better additive manufacturing grain crystallization can be obtained by reasonably adjusting the process parameter combinations. The transfer energy density can synthesize the influence of four key process parameters (*P*, *v*, the hatching distance (δ), and the layer thickness (*h*)). In this study, it is proposed that the **accepted energy density** will reflect the influence of five key process parameters, including the scanning trajectory (μ), which can reflect the comprehensive effect of process parameters more accurately.

## 1. Introduction

Additive manufacturing (AM) has a unique forming concept and several advantages; therefore, selective laser melting (SLM) is increasingly being used in small batches of parts that are difficult to machine [1]; however, because of the high requirements of SLM process parameter matching, it is difficult to directly control these parameters for better product performance [2]. In many production applications, the parameters need to be adjusted several times in order to make the necessary printed parts and, in some cases, manufacturing failure caused by inappropriate process sequences is also common. According to the literature [3], there are more than 130 process parameters that affect product performance, but there are five key parameters—laser power (*P*), scan speed (*s*), scanning strategy (μ), hatching distance (δ), and layer thickness (*h*). According to recent research, the mismatch of these process parameters forms an unbalanced temperature field, and the resulting large temperature gradient generates high thermal stress, resulting in corresponding defects such as air gaps [4], warpage [5], cracks [6], and geometric error [7], which lead to a decrease in the mechanical properties or to the deposition failure of the formed parts. Recently, there have been many reports on related process parameters and their corresponding mechanical properties, such as tensile properties [8], fatigue [9], hardness [10], surface roughness [11], and residual stress [12]. In our previous study [13], the influence of laser power and scanning strategy on residual stress distribution in 316L steel had been confirmed with a simulation combined experiment. It can be seen from the literature that the performance of the formed parts varies greatly under different combinations of processes.

The research on process parameters has now been developed from single parameter analyses to multi-parameter mixed analyses and the methods have been mainly to conduct experimental research on the process parameter group so as to analyze the microscopic and mechanical properties. Initially, the main research focused on a single parameter. Zheng et al. [14] found that with increasing applied laser power, the defects of the as-built parts were reduced significantly and the parts presented the highest relative density and tensile strength. In addition, Li et al. [15] found that the same energy density at different laser powers led to different phase formations, microstructures, textures, and mechanical properties for the fabricated selective laser melting. Wang et al. [16] indicated that an appropriate scan speed could result in a fine and stable microstructure that demonstrated a high hardness and tensile strength with a low elongation at break. Spierings et al. [17] analyzed the influence of varying laser scan speeds on the static mechanical properties of SLM processes, which increased the laser scan speed whereas the peak grain sizes in the fine-grained regions decreased. They also discussed the evolving microstructure and precipitation of nm-sized Al_3_Sc particles at different corresponding energy inputs. On the other hand, Lu et al. [18] studied the effect of the island scanning size on the microstructure and residual stress of In718. Jia et al. [19] analyzed the role of the scanning strategy on the residual stress distribution in a Ti-6Al-4V alloy prepared by SLM. Sg et al. [20] researched the influence of several specific scanning strategy parameters and the post Heat Treatment (HT) used upon the microstructure and the hardness of the SLM parts. Similar studies can be found in the literature. Hajnys et al. [21] designed three scanning strategies in simulation and verified these by experiment with samples which were inspired by the Bridge Curvature Method (BCM) shape. However, the conclusions of scanning trajectory research seem to be not so uniform.

Recently, some scholars focused on combining multiple process parameters for microscopic and mechanical property analysis. Simson et al. [22] studied the influence of energy density on the direction of residual stress under the action of multiple parameters. They measured the surface residual stress of a 316L sample generated by SLM with a lower energy density. In addition, in order to control the residual stress, a carefully planned scanning strategy was proposed. The appropriate process parameters (*P*, μ, and δ) were confirmed to play a fundamental role in determining the final properties (density, tensile, and fatigue behaviors) by Liverani et al. [23]. Teng et al. [24] found an AlSi10Mg alloy through batch experiments and obtained an optimum density (2.676 g/cm^3^) and Vickers hardness (115.92 HV) in the range of 48–63 J/mm^3^ laser energy density (LED). Bang et al. [25] achieved an energy density controlled between 9.34 and 23.98 J/mm^3^ to fabricate SUS316L parts with a high strength and high elongation characteristics using the SLM method. Huang et al. [26] fabricated two main types of SLM-316L HMDS samples (e.g., *P*, *s*, and *h*) that exhibited a typical layered morphology consisting of micron-sized melt pools and columnar grains. A lower hatch space resulted in a better density, elongation, and ultimate tensile strength for the SLM-316L HMDS when optimizing SLM process parameters. Lee et al. [27] investigated the effects of process parameters (e.g., *P*, *s*, δ, and *h*) on the high temperature strength of SLM 17-4PH stainless steel. The experiment verified that an energy density level of 64.29 J/mm^3^ better affected the microstructure, fractured surface, and cracking shape of the SP specimens. Ravichander et al. [28] studied the 20 suggested sets of SLM process parameters (*P*, *s*, and δ) in order to evaluate the dimensional accuracy, composition, and hardness corresponding to the interactions. The experiments tested a range of energy density values for the In718 superalloy needed to attain optimal values for each of the analyzed characteristics, and to enhance the fabrication quality of the as-built In718 specimens. Li et al. [29] systematically investigated the influence of each process parameter (including *P*, *s*, and δ) on the surface morphology, densification, and microstructure of tungsten samples. Pekok et al. [30] studied the effect of parameters (*P*, *s*, and δ) on the mechanical and microstructural properties of an as-fabricated aluminum 2024 alloy (AA2024) manufactured using SLM. They obtained a group of superior process parameter values through comparison. A similar study by Chen et al. [31], that researched the three factors and five levels of orthogonal experiments, was performed to validate the impact from process parameters (*P*, *s*, and δ) on steel-bronze bimetallic structures. Aqilah et al. [32] investigated the influence of process parameters (*P*, *s*, and δ) on the surface roughness of the specimens. It was found that laser power had the greatest effect on the surface roughness relative to the scanning speed and hatching distance.

From the above, it can be inferred that the selection of appropriate SLM parameters is crucial for determining the final material properties [33]. Either considering only the influence of a single factor, or mixing multiple parameters to conduct experiments, the performance influence relationship is relatively limited. Presently, experimental studies on the influence of process parameters on the deformation/stress of forming parts have not systematically considered the influence mechanism of each individual parameter. Therefore, the correlation between the influence of process parameters on performance cannot be clearly analyzed. Concurrently, the change of process parameters and its effect on the characteristics of the molten pool and the rule of stress field are even less studied; however, the process parameters directly affect the weld pool properties, temperature distribution, and residual stress, and influence its comprehensive mechanical properties in actual additive manufacturing [34].

Accordingly, the key process parameters of SLM forming are laser power, scanning speed, and scanning strategy, and in this study, we designed serialized process parameter experiments. The influence of the process parameters on the forming microstructure was analyzed, and the influence of these parameters on residual stress was further revealed. The aim of this study was to preliminarily explore multi-process parameter matching and process regularity. Additionally, this study offers some practical advice for successfully manufacturing parts using SLM.

## 2. Materials and Methods

### 2.1. Manufacturing Process

**Figure 1:**
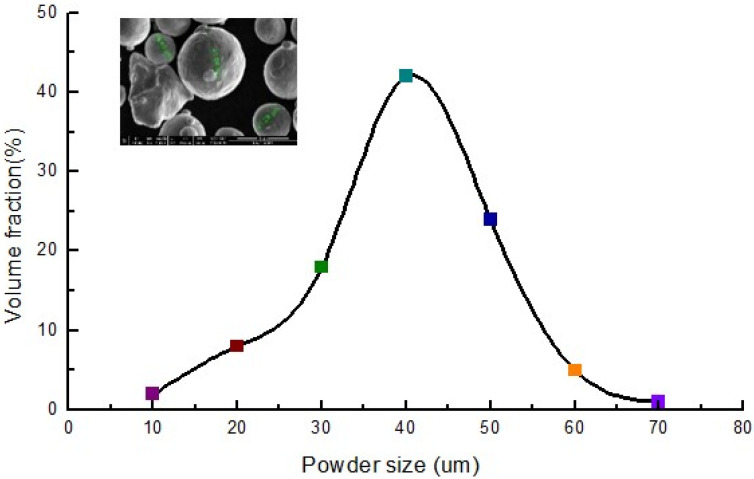
The 316L stainless steel powder.

**Figure 2:**
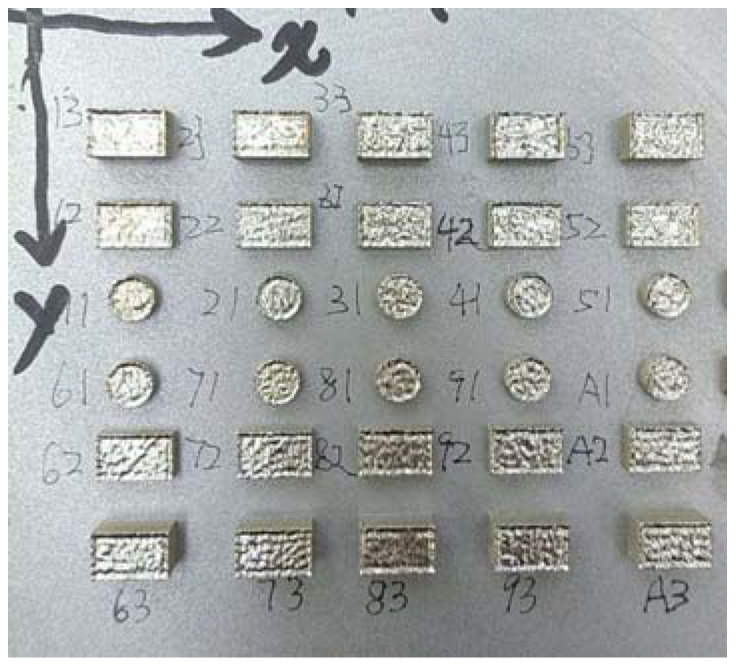
The 30 specimens fabricated by SLM 280. Numbers in the figure are the serial number of specimens, corresponds to Table 1 “Serial No.”.

Figure 1 shows the shape and the size distribution of the 316L stainless steel powder (SLM Solutions, Lübeck, Germany) utilized in this study. The size of most of the powder was about 40–50 μm in the volume distribution, as determined by statistical data method. There were 30 specimens that were fabricated using SLM 280 (SLM Solutions, Lübeck, Germany) equipped with a stainless steel baseplate, as shown in Figure 2. The main process parameters of SLM forming are shown in Table 1, which were designed according to the operating range of the S 280 equipment and experiential date. In this experiment, three main process parameters were considered: laser power (*P*), scanning speed (*s*), and scanning strategy (μ). A total of 10 groups in a small range orthogonal test samples with variable parameters were designed. In order to ensure the reliability of the follow-up measurement results, experiments were done in triplicate for each set of parameters; for example, the process parameters were the same for numbers 21, 22, and 23. One of them was used for microstructure testing, and two were used for residual stress testing. There were two kinds of structures for each group of parameters, with a sample size of 5 mm × 3 mm × 2 mm or Ф 4 mm × 2 mm. The other parameters were set as invariants as follows: layer thickness *h* = 50 μm, laser lap rate β = 10% or hatching distance δ = 0.1, laser absorption rate α = 0.7, and the initial temperature of the substrate was room temperature. The deposition samples’ relative density measurements were determined using the Archimedes principle. There has a high relative density from 97.6% to 99.2% against the theoretical value of 8.05 g/cm^3^ (316L) at room temperature [35].

**Figure 3:**
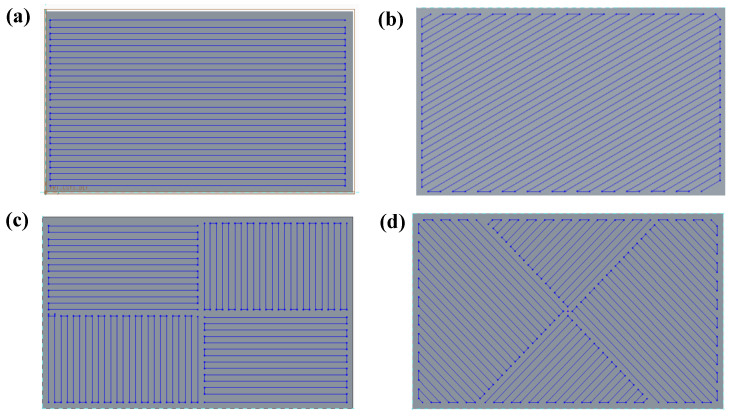
Laser scanning strategies for SLM: (**a**) stripe scanning strategy of 0°, (**b**) stripe scanning strategy of 30°, (**c**) chessboard scanning strategy of 0°, and (**d**) chessboard scanning strategy of 45°.

The two scanning paths that were adopted were the stripe scanning and chessboard scanning strategies, which are usually used in production, as shown in Figure 3. In the stripe scanning strategy where a 2/12 trajectory of a cycle was enumerated, such as in Figure 3a,b, the laser went from one end of the specimen to the other in a parallel line, folding back end to end. The deflection between layers was 30°. In this scanning mode, the influence time for before and after the molten pool became longer. The chessboard scanning strategy divided the work area into 2.5 mm^2^ square areas. Then, each area was scanned with a 45° stripe that varied with the layer. In this scanning mode where a 2/8 trajectory of a cycle was enumerated, such as in Figure 3c,d, the influence time for the front and rear molten pools was shorter. Comparing the two scanning strategies, the path of the laser was very different. As the scan direction and scan length were varied, the heat of the laser energy to be transferred to powder could be naturally changed [31]. This makes the manufacturability of SLM extremely challenging [30].

### 2.2. Test Methods

**Figure 4:**
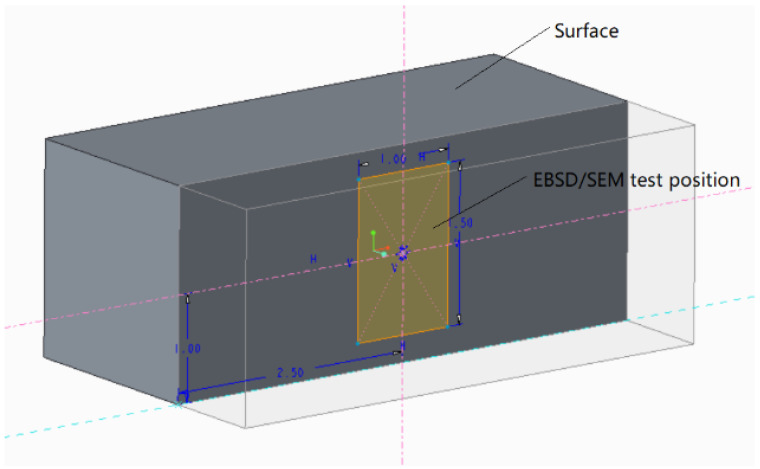
The test position of SEM and EBSD.

**Figure 5:**
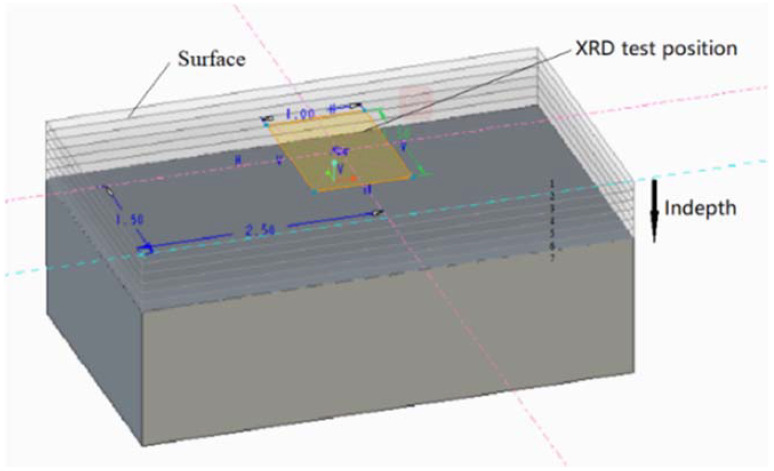
The test method for residual stress.

Figure 4 shows the experimental position and method of microstructure for SEM and EBSD. The test position was on the longitudinal section along the height. A scanning electron microscope (ZEISS Gemini500, Tokyo, Japan) was used to measure the microstructural changes of the molten pool at various parameters. Electron back scatter diffraction (FEI NanoSEM450, Hillsboro, OR, USA) was used to analyze the grain size and misorientation angle for various parameters. The residual stress test was performed using an X-ray diffraction (XRD) residual stress analyzer (Stresstech Group XSTRESS3000, Jyväskylä, Finland), as shown in Figure 5, which adopted an Mn-Target and a test deflection angle from 20° to 160°. The samples were directly tested after forming on the substrate in order to avoid stress release or change. The test locations were stripped from the surface to the bottom in a cross section, and a total of seven layers were tested. The saturated brine automatic etching method was adopted to strip and ensure consistency in the thickness (0.1 mm) of each layer. The X-ray scanning area was about 1 mm × 1.5 mm in the center of all samples.

## 3. Results

### 3.1. Molten Pool Morphology

**Figure 6:**
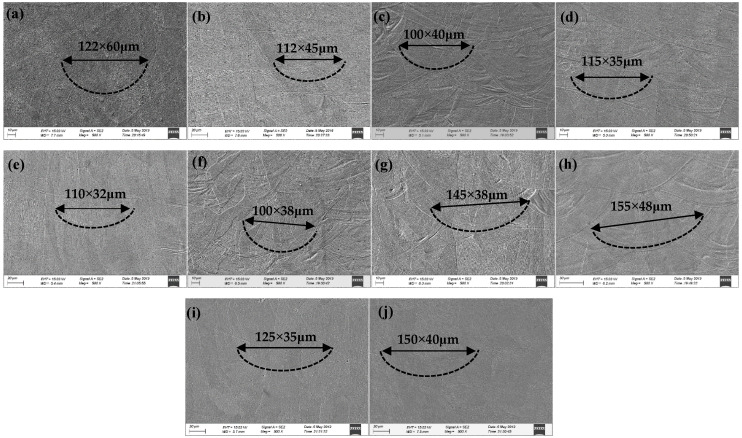
Geometric image analysis of the molten pool: (**a**) *P* = 160 W, *s* = 400 mm/s, striped; (**b**) *P* = 160 W, *s* = 500 mm/s, striped; (**c**) *P* = 160 W, *s* = 600 mm/s, striped; (**d**) *P* =160 W, *s* = 400 mm/s, chessboard; (**e**) *P* = 160 W, *s* = 500 mm/s, chessboard; (**f**) *P* = 160 W, *s* = 600 mm/s, chessboard; (**g**) *P* = 200 W, *s* = 800 mm/s, striped; (**h**) *P* = 200 W, *s* = 600 mm/s, striped; (**i**) *P* = 200 W, *s* = 600 mm/s, chessboard; and (**j**) *P* = 240 W, *s* = 800 mm/s, striped.

A microscopic section morphology of the 10 groups of samples with different process parameters was observed, and the section geometry is shown in Figure 6. The section profile of the molten pool was clearly demonstrated, which can reflect the superposition process of the molten pool layer by layer—the sample of the molten pool geometry was more consistent with the actual data. This could be seen from a comparative analysis of 10 groups of molten pool geometric data when using the three main process parameters (*P*, *s* and μ) as variables for the statistical analysis. First, in the case where only the power changed and other parameters were the same, the larger laser power caused a larger size of the molten pool (such as Figure 6c,g,j). Second, when the scanning speed changed, the slower scanning speed produced a larger geometric size for the molten pool (such as Figure 6a–c). Third, when the scanning strategy was compared, the molten pool size of the long scanning path (stripe) was larger than for those of the short scanning path (chessboard) (such as Figure 6a,d, Figure 6b,e, Figure 6h,i. All of the above rules could be obtained clearly from the comparison of the molten pool sizes in Figure 6a–j. These rules indicate that the combination of process parameters has a great influence on the features of the molten pool.

### 3.2. Grains and Orientation

**Figure 7:**
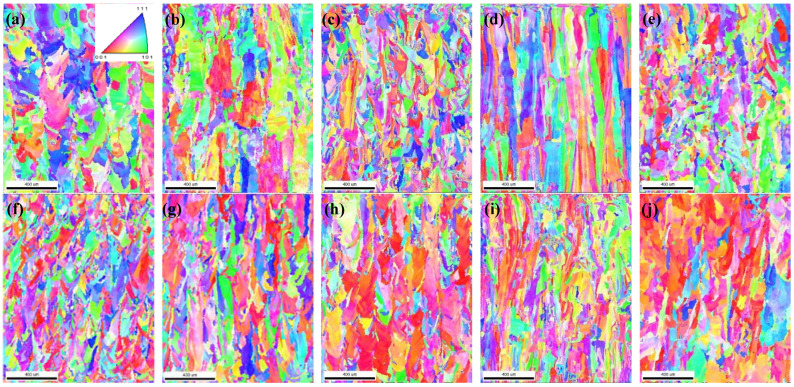
IPF diagram of EBSD: (**a**) *P* = 160 W, *s* = 400 mm/s, striped; (**b**) *P* = 160 W, *s* = 500 mm/s, striped; (**c**) *P* = 160 W, *s* = 600 mm/s, striped; (**d**) *P* = 160 W, *s* = 400 mm/s, chessboard; (**e**) *P* = 160 W, *s* = 500 mm/s, chessboard; (**f**) *P* = 160 W, *s* = 600 mm/s, chessboard; (**g**) *P* = 200 W, *s* = 800 mm/s, striped; (**h**) *P* = 200 W, *s* = 600 mm/s, striped; (**i**) *P* = 200 W, *s* = 600 mm/s, chessboard; and (**j**) *P* = 240 W, *s* = 800 mm/s, striped.

In addition, an EBSD test was carried out on the middle of the sample section at 1 mm × 1.5 mm. Figure 7 shows the microstructural changes with diverse shapes and different distributions of grain size on the 316L specimens corresponding to the 10 groups of process parameters chosen. The grain size was statistically analyzed in IPF maps. The grain size of the specimens fabricated by the largest power (240 W) and lowest scanning speed (400 mm/s) were relatively higher than for the other specimens. In addition, the grain size corresponding to the long scanning track (stripe) was slightly larger than for the short track (chessboard). Additionally, when these three parameters were matched in different ways, the grain growth mechanism was very different. As shown in Figure 7d,i, for the corresponding 160 W and 200 W power groups, the scanning speed was slow (400 mm/s) and the scanning trajectory was chessboard. The growth of the grains extended across the molten pool along the direction of the layer height in Figure 7d,i, and the crystallized grains grew in long strips, which was similar to the directional crystallization. From another point of view, as shown in Figure 7f of sample 6, with 160 W, matching the higher scanning speed of 600 mm/s and the short scanning track of the chessboard trajectory, the grain size of the sample was obviously the smallest compared with the other groups of process parameters.

**Figure 8:**
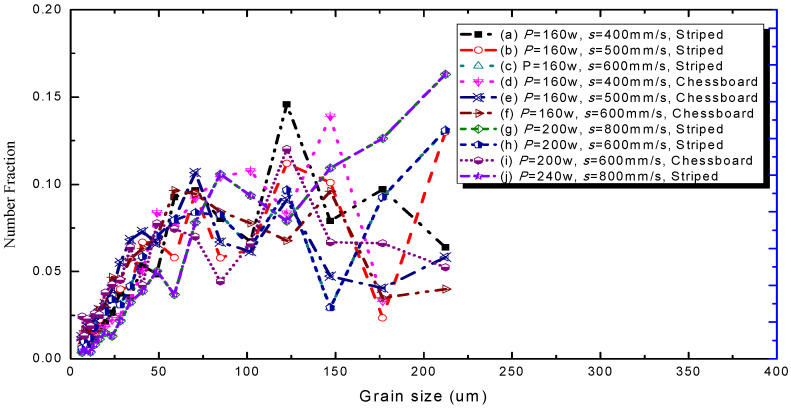
Grain size distribution diagram of the 10 samples.

**Figure 9:**
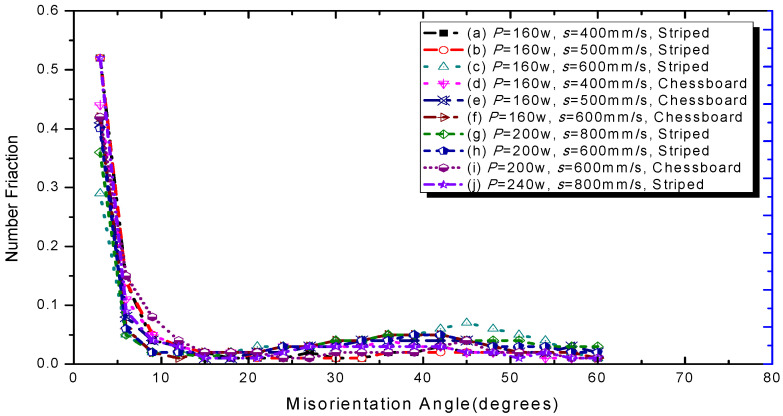
Misorientation angle statistical diagram of the 10 samples.

The grain size distribution diagram is shown in Figure 8. It can be seen that the smoother the curve is, the more uniform the grain size is. In addition, the grains of sample 6 are smaller and more uniform. The misorientation angle statistics corresponding to the IPF diagram are shown in Figure 9 with the 10 group process parameters, separately. It can be seen that most misorientation angles are distributed in small angles of less than 5°. As a result the laser moved faster in the molten pool, and the activity time of the metal atoms excited by heat was limited, for which the effect of rapid heating and cooling for the molten pool on the atoms had only a small angular deflection [36]. These results indicated that the control of the grain size and the morphology of the 316L part would be possible.

### 3.3. Residual Stress

According to the SLM forming process parameters in Table 1, the test results of the residual stress were divided into three ranks for comparison, as shown in Table 2. The first rank has different laser powers compared under the same parameters, for which there were three groups of data comparison results. The second rank was different scanning speeds compared under the same parameters, for which there were five groups of data. The third rank was the comparison of different scan trajectories, for which there were four groups of data when the other parameters were the same. The *X* coordinates of the graph in the table were spaced 0.1 mm inward from the 2.0 mm height position of the surface layer, and a total of seven measuring points were counted. The *Y* coordinate reflected the measured average value and its deviation of the residual stress of the current measured layer. In order to make the results universal, multiple data groups were obtained for each rank according to the trials. A comparison of the parameters of each group is made in Table 2. In addition, the grain size statistics of the corresponding sample in Figure 7 are also compared and listed in Table 2.

In order to facilitate a more intuitive comparison, the mean values of the stratified measurement of the residual stress corresponding to each group of process parameters were counted, which are listed in Table 3.

Table 2 shows the result curves, and Table 3 shows the average statistics. In the case of only different laser power, three power parameters were used (160 W and 200 W striped, 160 W and 200 W chessboard, and 200 W and 240 W striped). The higher laser power caused greater residual stress—the same trend was found for the three sets of parameters. In the case where the scanning speed was different, the slower scanning speed caused a greater residual stress after condensation; this trend was shared by the five groups of curves. The residual stress of the striped scanning was larger than that of the chessboard scanning when only the scanning trajectory was compared. Therefore, the residual stress of the specimen with less power, higher speed, and chessboard scanning was the lowest when the parameters satisfied the forming quality condition (e.g., sample 6). It should also be noted that the sample with a low residual stress had a smaller grain size. These data show that the above three key process parameters have a significant impact on the residual stress results. The residual stress could also be controlled by adjusting the process parameters [37].

## 4. Discussion

As the laser power and the scanning speed were varied, the energy density imposed on the powder could be naturally changed [38]. For the molten pool formed after laser action on the powder material, the important parameter of laser energy density was introduced to reflect the result of the interaction between the laser energy and material. The transferred energy density *E* (J/mm^3^) was calculated as follows:

(1) E(t)=P·αs·δ·h=Wmm3s=Jmm3

where *P* (W) is the laser power, *s* (mm/s) is the scanning speed, δ (mm) is the hatching distance, *h* (mm) is the effective layer thickness given for the laser energy, and α is the laser absorption rate. The effective layer thickness given for laser energy was assumed to be the sum of the layer thickness of 50 µm and the hatch distance was considered to be 0.1 mm.

In Equation (1), only four main process parameters were considered. A constant energy density with varying laser parameters results in considerable differences in the forming property [39]. It can be seen from the above experimental data that the scanning trajectory also had a significant influence on the obtained properties. Therefore, the laser-transferred energy density needed to be revised to the accepted energy density of the workpiece.

(2) E(a)=P·αs·δ·h·μ=Wmm3s=Jmm3

where μ is the scanning strategy mode coefficient. It is related to the length of the scanning path, the deflection angle, the trajectory cycle, etc., so we will aim to continue searching for the determination method of its coefficient value in the future research. The two scanning strategies in this paper could preliminarily attempt to define two coefficients separately, which are 1.1 for μ_1_ in the striped scanning and 1 for μ_2_ in the chessboard scanning to be tried. In this way, the actual difference effect of the laser energy could be more reflected in the experimental results.

The average molten pool size, grain size, and residual stress in the different process parameters are listed in Table 4. The relevant laws could be analyzed through the statistical results as follows.

From the molten pool morphology of the formed SLM, the larger laser power and the lower scanning speed formed a higher laser energy density and produced a larger molten pool geometry in the SLM forming process. Corresponding to the longer solidification time, the metal atoms could be combined to crystallize a longer time, thus forming a larger grain size. At the same time, the influence of the scanning trajectory was also very obvious. The grain size depended on the track length when the energy flow density was the same. The conclusion was that the shorter the scanning track, the smaller the crystallized grain.

**Figure 10:**
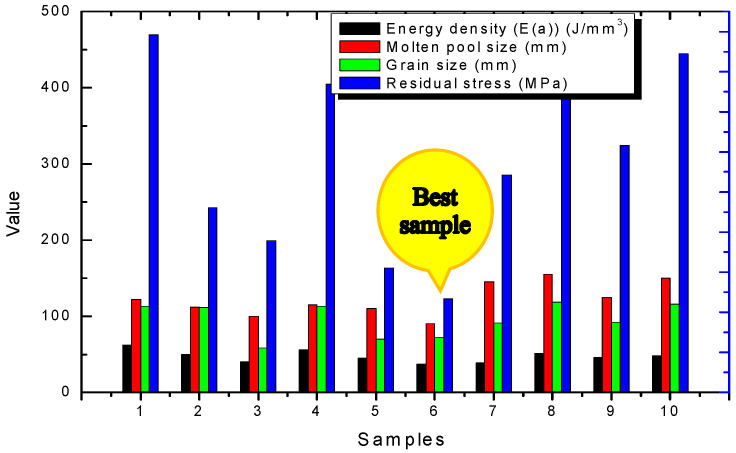
Histogram of the relationship between the performance parameters and laser energy flow density.

In addition, as can be seen from the grain size statistics of the corresponding parameters in Table 4, the sample with a larger grain size took up a larger proportion of the residual stress, corresponding to the sample under the comprehensive action of the process parameters, and vice versa. It can be seen that under the action of technological parameters, such as high power, high temperature of the molten pool, and slow relative cooling rate, the metal atoms were formed and the movement of the metal atoms was intensified when heated, which resulted in easy growth of molten metal grains. At the same time, the grains grew across the molten pool, and the heat of the metal atoms deviating from the equilibrium position increased, forming a large residual stress. The relationship between the corresponding performance parameters and the laser energy density was visually represented by a histogram, as shown in Figure 10.

The matching of process parameters was particularly important. Under a certain laser power and a smaller scanning speed, the molten pool was larger, which caused the heat-affected zone to expand, and the directional growth of columnar grains [26] had better unidirectional mechanical properties along the vertical direction. The residual stress was found to be large too, such as in samples 4 and 9. When a certain laser power was combined with a larger scanning speed, the molten pool became smaller and the crystalline grains were also very small as a result of rapid cooling, but they had a good microstructure and less residual stress, as seen in sample 6. It was the “best sample” in all these experimental samples. Additionally, its bimodal structure was evident, as shown in Figure 10. Meanwhile, it had the lowest accepted energy density as shown in Figure 10 in combination with Table 4. It can be seen that when the powder material can be fused, the smaller accepted laser energy density could beneficial to the forming thermodynamics field.

## 5. Conclusions

In this study, the molten pool geometry, grain size, misorientation angle, and the corresponding residual stress of the formed specimens were tested by designing 10 groups of process parameters with different gradients. It was proven that the process parameters directly affect the morphology and size of the molten pool in the SLM deposition, and the molten pool has a direct effect on the grain size and crystal orientation distribution. In addition, the grain size and orientation distribution also affect the value of the residual stress. Therefore, better additive manufacturing grain crystallization can be obtained by reasonably adjusting the process parameter combinations. At the same time, some basic regulation rules of process parameters were obtained.

(1) Laser power has the main effect on the microstructure. As the power increases, the molten pool increases significantly and the crystalline grain is larger, which also causes a larger thermal stress and residual stress in a case where the other parameters remain unchanged.
(2) The scanning speed also has a great influence on the microstructure of the forming part. The lower the scanning speed is, the longer the heating time of the molten pool, which leads to a larger geometrical size and crystallization and to an increase in residual stress when the other parameters remain the same.
(3) The scanning mode, i.e., the length of the scanning track, also has a significant effect. In general, a short scanning trajectory has better organization and performance indicators.
(4) The matching and coordination of the process parameters have a significant influence on the microstructure and mechanical properties of SLM forming, which can be evaluated by the accepted energy density. It was confirmed that reasonable matching of the relationship between the process parameters can obtain excellent forming properties, e.g., grain refinement and columnar grain, which presented as approximately directional solidification.

## Figures and Tables

**Table 1:** The main process parameters of the SLM specimens.

Samples	Laser Power	Scanning Speed	Scanning Strategy	Hatching Distance	Layer Thickness	Serial No.
1	*P* = 160 W	*s* = 400 mm/s	striped	0.1 mm	50 μm	11, 12, 13
2	*P* = 160 W	*s* = 500 mm/s	striped	0.1 mm	50 μm	21, 22, 23
3	*P* = 160 W	*s* = 600 mm/s	striped	0.1 mm	50 μm	31, 32, 33
4	*P* = 160 W	*s* = 400 mm/s	chessboard	0.1 mm	50 μm	41, 42, 43
5	*P* = 160 W	*s* = 500 mm/s	chessboard	0.1 mm	50 μm	51, 52, 53
6	*P* = 160 W	*s* = 600 mm/s	chessboard	0.1 mm	50 μm	61, 62, 63
7	*P* = 200 W	*s* = 800 mm/s	striped	0.1 mm	50 μm	71, 72, 73
8	*P* = 200 W	*s* = 600 mm/s	striped	0.1 mm	50 μm	81, 82, 83
9	*P* = 200 W	*s* = 600 mm/s	chessboard	0.1 mm	50 μm	91, 92, 93
A	*P* = 240 W	*s* = 800 mm/s	striped	0.1 mm	50 μm	A1, A2, A3

**Table 2:** Comparison of the results for the different parameters of the SLM specimens.

Rank	Different Laser Power	Different Scanning Speed	Different Scan Trajectories
1	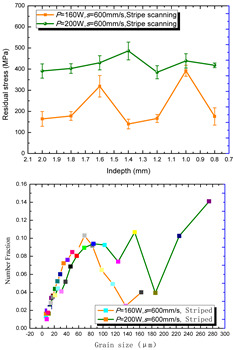	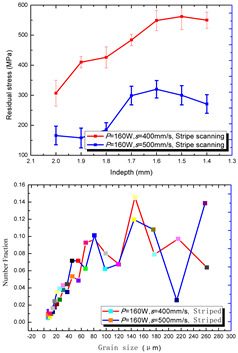	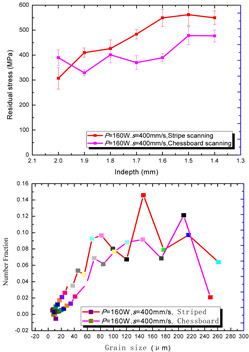
2	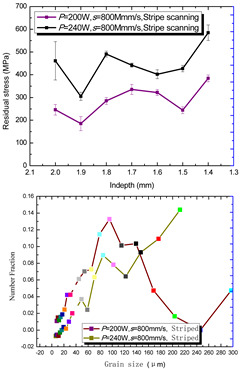	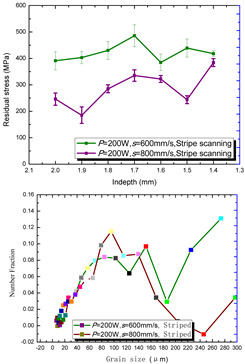	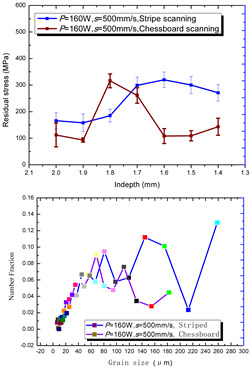
3	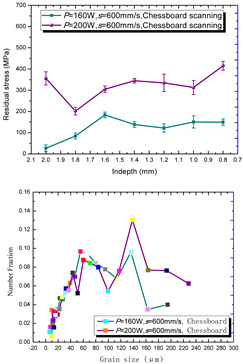	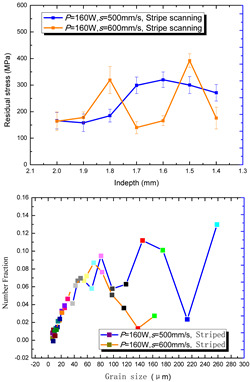	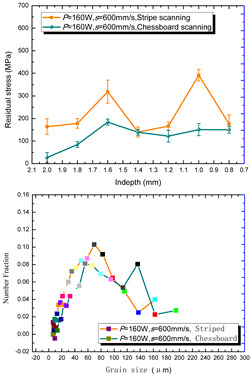
4	————————	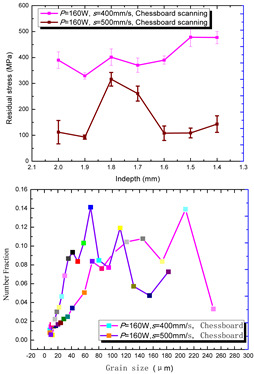	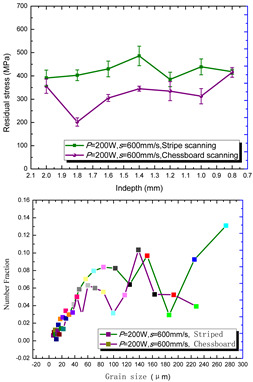
5	————————	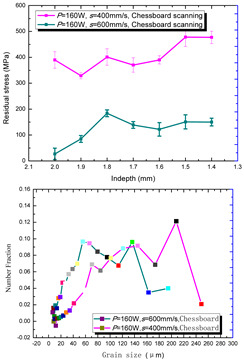	————————

**Table 3:** The average values of residual stress in different groups (MPa).

Method	Group 1		Group 2		Group 3		Group 4		Group 5	
* Power comparison *	*s* = 600 mm/s;		*s* = 800 mm/s; Striped scanning		*s* = 600 mm/s; Chessboard scanning		------		------	
	*P* = 200	*P* = 160	*P* = 240	*P* = 200	*P* = 200	*P* = 160				
	421.7	199.3	444.7	285.8	324.4	122.6				
* Speed comparison *	*P* = 160 W; Striped scanning		*P* = 160 W; Striped scanning		*P* = 200 W; Striped scanning		*P* = 160 W; Chessboard scanning		*P* = 160 W; Chessboard scanning	
	*s* = 400	*s* = 500	*s* = 500	*s* = 600	*s* = 600	*s* = 800	*s* = 400	*s* = 500	*s* = 400	*s* = 600
	469.7	242.7	242.7	199.3	421.7	285.5	405	163.2	405	122.6
* Different scanning strategy *	*P* = 160 W; *s* = 400 mm/s		*P* = 160 W; *s* = 600 mm/s		*P* = 200 W; *s* = 600 mm/s		*P* = 160 W; *s* = 500 mm/s		------	
	Striped	Chessboard	Striped	Chessboard	Striped	Chessboard	Striped	Chessboard		
	469.7	405	199.3	122.6	421.7	324.4	242.7	163.2		

**Table 4:** The average molten pool size, grain size, and residual stress in the different parameters.

Sample	Power	Scanning Speed	Scanning Strategy	Energy Density (*E*(t))	Energy Density (*E*(a))	Molten Pool (mm)	Grain Size (mm)	Residual Stress (MPa)
1	160 W	400 mm/s	Striped	56	62	122 × 60	112.9	469.7
2	160 W	500 mm/s	Striped	45	50	112 × 45	111.6	242.7
3	160 W	600 mm/s	Striped	37	40	100 × 40	58.1	199.3
4	160 W	400 mm/s	Chessboard	56	56	115 × 35	113.1	405
5	160 W	500 mm/s	Chessboard	45	45	110 × 32	69.9	163.2
6	160 W	600 mm/s	Chessboard	37	37	100 × 38	72.1	122.6
7	200 W	800 mm/s	Striped	35	39	145 × 38	91.3	285.5
8	200 W	600 mm/s	Striped	46	51	155 × 48	118.8	421.7
9	200 W	600 mm/s	Chessboard	46	46	125 × 35	91.7	324.4
A	240 W	800 mm/s	Striped	42	48	150 × 40	115.5	444.7

## Data Availability

The datasets used and analyzed during this study are available from the corresponding author on reasonable request.
